# The emerging role of Toll-like receptor 4 in myocardial inflammation

**DOI:** 10.1038/cddis.2016.140

**Published:** 2016-05-26

**Authors:** Y Yang, J Lv, S Jiang, Z Ma, D Wang, W Hu, C Deng, C Fan, S Di, Y Sun, W Yi

**Affiliations:** 1 Department of Cardiovascular Surgery, Xijing Hospital, The Fourth Military Medical University, 127 Changle West Road, Xi'an 710032, China; 2 Department of Thoracic and Cardiovascular Surgery, Affiliated Drum Tower Hospital of Nanjing University Medical School, 321 Zhongshan Road, Nanjing, Jiangsu 210008, China; 3 Department of Biomedical Engineering, The Fourth Military Medical University, 169 Changle West Road, Xi'an 710032, China; 4 Department of Aerospace Medicine, The Fourth Military Medical University, Xi'an 710032, China; 5 Department of Thoracic Surgery, Tangdu Hospital, The Fourth Military Medical University, 1 Xinsi Road, Xi'an 710038, China; 6 Department of Geriatrics, Xijing Hospital, The Fourth Military Medical University, 127 Changle West Road, Xi'an 710032, China

## Abstract

Toll-like receptors (TLRs) are a family of pattern recognition receptors involved in cardiovascular diseases. Notably, numerous studies have demonstrated that TLR4 activates the expression of several of pro-inflammatory cytokine genes that play pivotal roles in myocardial inflammation, particularly myocarditis, myocardial infarction, ischemia-reperfusion injury, and heart failure. In addition, TLR4 is an emerging target for anti-inflammatory therapies. Given the significance of TLR4, it would be useful to summarize the current literature on the molecular mechanisms and roles of TLR4 in myocardial inflammation. Thus, in this review, we first introduce the basic knowledge of the *TLR4* gene and describe the activation and signaling pathways of TLR4 in myocardial inflammation. Moreover, we highlight the recent progress of research on the involvement of TLR4 in myocardial inflammation. The information reviewed here may be useful to further experimental research and to increase the potential of TLR4 as a therapeutic target.

## Facts

Human TLR4 was the first mammalian Toll protein to be characterized and is secreted from the endoplasmic reticulum.
TLR4 initiates the expression of a number of pro-inflammatory genes, cell surface molecules, and chemokines through the MyD88-dependent pathway.
TLR4 performs a wide variety of functions in various pathological conditions, including cardiovascular disease, allergic diseases, obesity-associated metabolic diseases, neuronal degeneration, apoptosis, autoimmune disorders, infectious diseases, and inflammatory bowel diseases.

## Open Questions

What is the exact molecular mechanism of TLR4 in myocardial inflammation?
How is TLR4 related to diagnosis and prognosis of diseases related to myocardial inflammation?
Which therapeutic strategy would be the most effective against TLR4-associated myocardial inflammation in clinical settings?

Myocardial inflammation has been widely accepted to play a pivotal role in the physiological and pathological mechanisms of cardiac function and dysfunction. Efficient inflammation is required for host defense against damage and tissue repair. However, excessive or chronic myocardial inflammation, which is reported to induce severe damage to the myocardium, is involved in a number of cardiac disorders, such as myocarditis,^1^ myocardial infarction (MI),^2^ ischemia-reperfusion (I/R) injury,^3^ heart failure,^4^ aortic valve diseases,^5^ atherosclerosis,^6^ and hypertension.^7^ During myocardial inflammation, cardiac myocytes express various molecules that contribute to the infiltration of neutrophils into the myocardium, including pro-inflammatory cytokines, cell surface molecules, and chemokines. The regulatory mechanisms of myocardial inflammation are multifaceted. The host inflammation is initiated by pattern recognition receptors (PRRs), which are essential components of the innate immune system.^8^ PRRs recognize and respond to damage-associated molecular patterns (DAMPs), including exogenous pathogen-associated molecular patterns (PAMPs) that are conserved structures of the pathogenic micro-organisms and endogenous alarmins that are released in response to stress or tissue damage.^9^

Among PRRs, Toll-like receptors (TLRs) were first described and have been studied most intensively. Engagement of TLRs by DAMPs activates inflammatory responses of cardiac myocytes, which represents the first line of innate host defense and modulates the adaptive immune responses.^10^ TLR4, a key member of the TLRs, has been reported to perform a wide variety of functions in various pathological conditions, including cardiovascular disease,^4^ allergic diseases,^11^ obesity-associated metabolic diseases,^12^ neuronal degeneration,^13^ apoptosis,^14^ autoimmune disorders,^15^ infectious diseases,^16^ and inflammatory bowel diseases.^17^ Most importantly, TLR4, whose levels are the highest compared with other TLRs in the heart, plays a critical role in myocardial inflammation, including myocarditis,^18^ MI,^19^ I/R injury,^20^ heart failure,^4^ aortic valve diseases,^21^ atherosclerosis,^22^ and hypertension.^23^

In this review, we present the elaborate network of roles that TLR4 plays in myocardial inflammation. First, we introduce the basic knowledge of TLR4. We then describe the activation and signaling pathways of TLR4 in myocardial inflammation. Finally, we highlight the involvement of TLR4 in myocardial inflammation, including myocarditis, MI, and I/R injury, and the clinical prospective of TLR4 inhibition. This review presents a comprehensive picture of the roles that TLR4 plays in myocardial inflammation and may contribute to the promotion of TLR4 as a new therapeutic target.

## General Background on TLRs

TLRs are a family of PRRs initially identified as Toll proteins in 1984 for their roles in the early embryogenesis of the fruit fly *Drosophila melanogaster*.^24^ TLRs are expressed in various types of heart cells, including cardiac myocytes, smooth muscle cells, and endothelial cells.^25^ The relative expression levels for TLR messenger RNAs (mRNAs) in the human heart rank as TLR4 > TLR2 > TLR3 > TLR5 > TLR1 > TLR6 > TLR7 > TLR8 > TLR9 > TLR10. Notably, the relative mRNA expression for TLR2, TLR3, or TLR4 is ~10-fold higher than TLR1 or TLR5–10.^26^ In addition, TLR expression has also been observed in brain, kidney, liver, lung, small intestine, spinal cord, spleen, and reproductive organs.^27^

Although extracellular and endosomal TLRs have similar ectodomain sequences, they provide a platform for the recognition of a different set of TLR ligands respectively. One mechanism of ligand discrimination is dependent on the different ectodomain residues of distinct TLRs. The leucine-rich repeat motifs located in the ectodomains of TLRs consist of 20–30 amino acids including the consensus sequence LxxLxLxxN. Amino acid variations within these motifs lead to variations in structural conformation that allows for ligand interaction.^28^ Another mechanism relies on the formation of homodimers and heterodimers. Most TLRs form homodimers, with a few exceptions. For instance, TLR2 forms heterodimers with TLR1 or TLR6, thus enabling differential recognition of lipopeptides: TLR1–TLR2 recognizes triacylated lipopeptides, whereas TLR2-TLR6 recognizes diacylated lipopeptides.^29, 30^ Besides, cofactors can also reflect the diversity of TLR ligand composition, since they ensure proper detection of DAMPs and discrimination between self and non-self. Take TLR4 for an example, TLR4 homodimers bind to cofactors, such as CD14, myeloid differentiation factor 2 (MD2), and lipopolysaccharide (LPS)-binding protein (LBP), which can further deliver particular molecules while avoiding other ligands. To ensure TLR4 can reach the assigned subcellular compartments to bind to ligands and initiate signaling, these cofactors also play roles in proper folding of TLR4 in the endoplasmic reticulum (ER), localization to the appropriate subcellular compartment, and protein processing.^31^

Interestingly, the risk for excessive inflammation is particularly low over the first few years of life but rises with advancing age. Accumulating evidence has suggested that TLR sensor function is well developed in newborns. The expression of TLRs and their downstream targets in mononuclear cells of healthy infants appears to be stable at adult-like levels over the first 5 years of life. In other words, differential TLR expression appears unlikely to be a main cause for the altered susceptibility to inflammation in early versus adult life. Although TLR expression and signaling in early life appear similar to those in adults, TLR-mediated production of innate immune effectors is significantly decreased in early life. Virtually, TLR4 mostly confers an anti-inflammatory effect on the hearts of newborns via high production of IL-10, whereas all other innate responses are low. In older individuals, TLR4 is generally expressed in monocytes at lower levels. In addition, older adults may exhibit a decrease in nearly all innate TLR-induced responses and higher basal levels of many pro-inflammatory cytokines.^32^

## Characterization of TLR4

Human TLR4 was the first mammalian Toll protein to be characterized,^33^ and it is secreted from the ER. In association with its chaperone molecules, coreceptors, and adapter proteins, TLR4 is trafficked to the cis-Golgi in a coat protein complex II-coated vesicle. Subsequently, TLR4 is exported to the plasma membrane, where it responds to its ligands and triggers a series of inflammatory cascades.^14^ TLR4 is activated by LPS, which involves the cofactors CD14, MD2, and LBP. TLR4 interacts with LBP to form a complex that is recognized by CD14. CD14 is a protein that binds LBP and delivers LPS-LBP to the TLR4-MD2 complex on the cell surface.^31, 34, 35^ In addition, TLR4 is also involved in the recognition of exogenous ligands PAMPs, such as the fusion protein from respiratory syncytial virus and glycerophosphatidylinositol anchors from parasites. Endogenous ligands alarmins, such as heat shock protein, high mobility group box 1 (HMGB1), the extra domain A of fibronectins, oligosaccharides of hyaluronic acid, heparan sulfate, and fibrinogen, are released in response to tissue injury and further activate TLR4 thereby triggering the innate immune system (Table 1).^36, 37^ Recent research suggests that macrophage scavenger receptor class A (SR-A) is a co-receptor for TLR4 to facilitate inflammatory responses, suppress cell survival, and promote cell apoptosis.^38^ However, further studies are needed to determine how TLR4 interacts with SRs during myocardial inflammation.

Growing amounts of data suggest that the ability of certain individuals to respond properly to TLR4 ligands may be impaired by single nucleotide polymorphisms (SNPs) within the *TLR4* gene, resulting in an altered susceptibility to infectious and inflammatory diseases. Among them, two cosegregating SNPs in *TLR4* have been studied the most extensively: Asp299Gly and Thr399Ile.^39^ Ameziane *et al.*^40^ have revealed that the Asp299Gly polymorphism of the human TLR4 receptor has a protective effect on acute coronary events. This is confirmed by Balistreri *et al.*^41^ that have found patients with Asp299Gly have a reduced risk of MI. The underlying mechanism may involve the altered composition and structure of TLR4 ectodomain by the Asp299Gly polymorphism, leading to a decrease in the interaction of TLR4 with DAMPs and thereby attenuated TLR4-mediated myocardial inflammation. However, clinical studies that have attempted to link the risk of myocardial inflammation with *TLR4* polymorphisms have yielded inconsistent results. The largest study involving nearly 5000 individuals has shown no association between the *TLR4* Asp299Gly polymorphism and MI.^42^ Interestingly, Edfeldt *et al.* have found that Asp299Gly and Thr399Ile *TLR4* genotypes exhibit an increased risk of MI in men, instead of women.^43^ Therefore, studies regarding the association of *TLR4* genetic variants with susceptibility to myocardial inflammation remain conflicting. This may be attributed to a simplistic view of these haplotypes, as revealed by the possibility to inherit both Asp299Gly and Thr399Ile polymorphisms.^44^ All of these studies are still faced with challenges due to wide range of allele frequencies possibly caused by population and ethnic differences in the control groups, making replication studies more difficult.^26^ Hence, the roles of *TLR4* genotypes in myocardial inflammation require to be further clarified.

## TLR4 Signaling Pathways

The activation of TLR4 by LPS induces two signaling pathways: the myeloid differentiation factor 88 (MyD88)-dependent and the MyD88-independent pathways. These signaling pathways activate many transcription factors, such as nuclear factor-*κ*B (NF-*κ*B) and interferon-regulatory factors (IRFs), and subsequently induce the production of pro-inflammatory cytokines and interferons (IFNs), respectively. Remarkably, a recent study has shown that TLR4 and some other signaling pathways, such as the phosphatidylinositol 3-kinase (PI3K)/Akt (also known as protein kinase B) signaling pathway counter-regulate each other.^45, 46, 47^ However, the precise regulatory mechanisms are still unknown. Herein, we mainly present the elaborate network of the two classical TLR4 signaling pathways.

### The MyD88-dependent pathway

**Figure 1:**
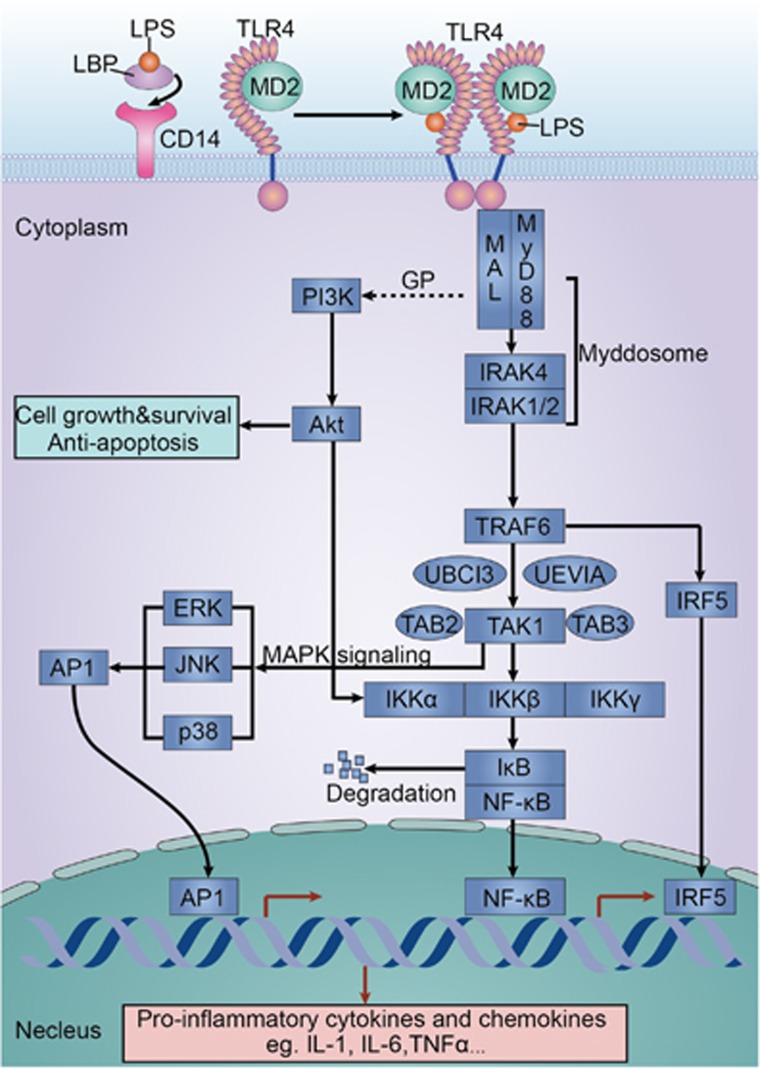
MyD88-dependent pathway. TLR4 uses CD14 to respond to LPS. LBP binds to LPS and presents it to CD14. MD2 is necessary for TLR4 to bind to LPS. MAL connects TLR4 and MyD88. Afterwards, MyD88 binds to IRAK4 and IRAK1/2. The IRAK complex then dissociates from MyD88 and interacts with TRAF6. TRAF6 forms a complex with TAK1, TAB1, and TAB2. Later, the complex binds to ubiquitin ligases, including UBC13 and UEV1A. TAK1 then activates the IKK*α*/IKK*β*/IKKγ complex and induces I*κ*B phosphorylation. Phosphorylated I*κ*B dissociates from the complex and is directly targeted for ubiquitination and degradation by proteasomes, thus activating the transcription factor NF-*κ*B. The released NF-*κ*B translocates into the nucleus and mediates the expression of a number of pro-inflammatory cytokine genes. In addition to the activation of the IKK complex, TAK1 can activate MAPK signaling pathways, such as ERK, JNK, and the p38 pathway. MAPK signaling pathways can activate the transcription factor AP-1. Activation of NF-*κ*B and AP-1 contributes to the expression of pro-inflammatory cytokines, such as IL-1, IL-6, and TNF-*α*. Notably, GP leads to TLR4 disassociating from MyD88, binding PI3K, further phosphorylating Akt and promoting survival and inhibits cardiac myocyte apoptosis. Gene names: ERK, extracellular signal-regulated kinase; GP, glucan phosphate; IKK, inhibitory *κ*B (I*κ*B) kinase; JNK, c-Jun N-terminal kinase; MAL, MyD88-adapter like; UBC13, ubiquitin-conjugating enzyme 13; UEV1A, ubiquitin-conjugating enzyme variant 1A

The MyD88-dependent pathway is initiated via MyD88 after TLR4 activation. MyD88, an adapter protein with a death domain, is crucial for TLR4 signaling pathways. MyD88-adapter like (also known as Toll/interleukin-1 receptor (TIR) domain-containing adapter protein) connects the cytoplasmic TIR domain of TLR4 and MyD88. Afterwards, the death domain of MyD88 binds to the IL-1 receptor-associated kinase 4 (IRAK4), which activates one of the other IRAK family members, IRAK1 or IRAK2.^48, 49^ The complex of the MyD88-IRAKs family has been called the ‘Myddosome' and is especially essential for inflammation and the host immune response.^50^ The IRAK complex then dissociates from the Myddosome and interacts with tumor necrosis factor (TNF) receptor-associated factor 6 (TRAF6). TRAF6 forms a complex with transforming growth factor-*β*-activated kinase 1 (TAK1), TAK1-binding protein 1 (TAB1), and TAB2. Afterwards, the complex binds to ubiquitin ligases, including ubiquitin-conjugating enzyme 13 and ubiquitin-conjugating enzyme variant 1A. TAK1 then activates the complex of inhibitory *κ*B (I*κ*B) kinase *α* (IKK*α*)/IKK*β*/IKK*γ* (also known as IKK1, IKK2, and NEMO, respectively) and induces I*κ*B phosphorylation. Phosphorylated I*κ*B dissociates from the complex and is directly targeted for ubiquitination and proteasomal degradation, thus activating the transcription factor NF-*κ*B. The released NF-*κ*B translocates into the nucleus and mediates the expression of a number of pro-inflammatory cytokine genes.^51, 52^ In addition to the activation of the IKK complex, TAK1 can activate mitogen-activated protein kinase (MAPK) signaling pathways, such as the extracellular signal-regulated kinase pathway, the c-Jun N-terminal kinase pathway, and the p38 pathway.^53^ MAPK signaling pathways can activate the transcription factor activator protein-1 (AP-1). The activation of NF-*κ*B and AP-1 contributes to the expression of pro-inflammatory cytokines, such as IL-6, IL-1, and TNF-*α* (Figure 1).^54^

### The MyD88-independent pathway

The MyD88-independent pathway, also known as the TIR-domain-containing adapter-inducing IFN-*β* (TRIF)-dependent pathway or the TIR-containing adapter molecule-1-dependent pathway, can lead to the activation of both IRF and NF-*κ*B and the subsequent expression of IFNs and pro-inflammatory cytokines, respectively.

**Figure 2:**
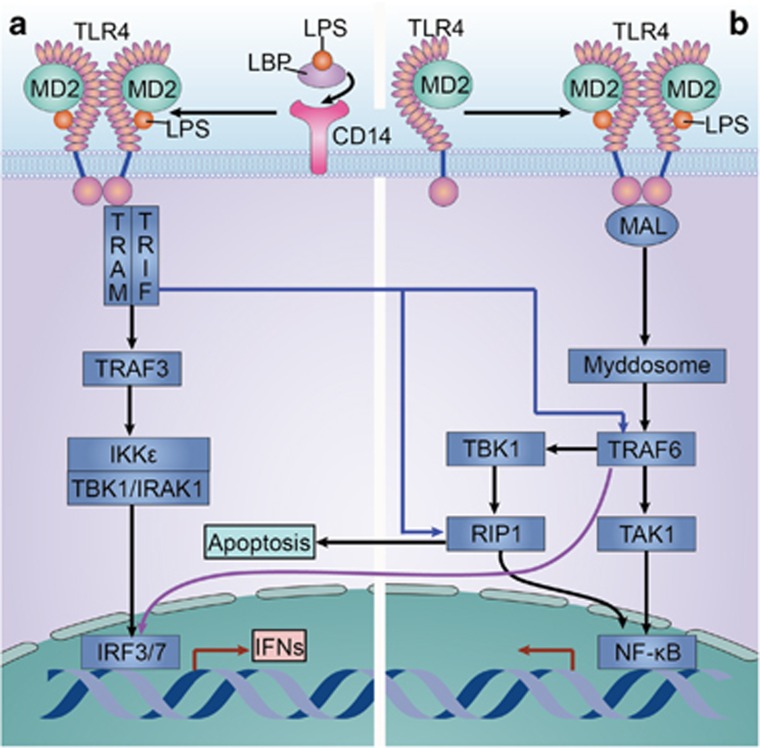
(**a**) The MyD88-independent pathway. (**b**) Crosstalk between the MyD88-dependent and MyD88-independent pathways. The MyD88-independent pathway can lead to the activation of both IRF and NF-kB and the subsequent expression of IFNs and pro-inflammatory cytokines, respectively. The MyD88-independent pathway is initiated by TRAM and TRIF. After being recruited, TRIF interacts with TBK and IKK-*ɛ* to phosphorylate the transcription factor IRF3. TRIF can also interact with IRAK1 and IKK-*ɛ* to activate the transcription factor IRF7. Activated IRFs then translocate into nucleus, bind with DNA, and produce such antiviral molecules as IFN-*β*. In addition, TRIF can also promote MyD88-independent NF-*κ*B activation in TLR4 signaling pathways. Similar to the MyD88-dependent pathway, TRIF recruits TRAF6 and activates TAK1 through ubiquitination-dependent mechanisms, which in turn activates the NF-*κ*B and MAPK pathways. Moreover, TRIF also activates MyD88-independent NF-*κ*B activation by recruiting RIP1. Thus, TRIF activates IRFs by interacting with TBK1 and IRAK1 and activates NF-*κ*B by interacting with RIP1. Furthermore, RIP1 can interact with FADD, which initiates caspase cascades and then induces cell apoptosis. Gene names: IKK-*ɛ*, inhibitory *κ*B (I*κ*B) kinase *ɛ*; IRAK1, interleukin (IL)-1 receptor-associated kinase 1; IRF, IFN-regulatory factor; TANK, TRAF family member-associated NF-*κ*B activator; TRIF, TIR-domain-containing adapter-inducing interferon-*β*; TLR4, Toll-like receptor 4

The MyD88-independent pathway is initiated by the TRIF-related adapter molecule (TRAM) and TRIF. TRAM is a particular bridging adapter protein in the TLR4-mediated MyD88-independent pathway, which is localized to the cytosolic surface of the plasma membrane.^14^ After being recruited, TRIF interacts with TRAF family member-associated NF-*κ*B activator-binding kinase 1 (TBK1) and IKK-*ɛ* for the phosphorylation of the transcription factor IRF3.^55^ TRIF can also interact with IRAK1 and IKK-*ɛ* for the activation of the transcription factor IRF7. Activated IRFs then translocate into nucleus, bind with DNA, and produce such antiviral molecules as IFN-*β*.^51, 56^ In addition, TRIF can also promote MyD88-independent NF-*κ*B activation in TLR4 signaling pathways. Similar to the MyD88-dependent pathway, TRIF recruits TRAF6 and activates TAK1 through ubiquitination-dependent mechanisms, which in turn activates the NF-*κ*B and the MAPK pathways.^57^ Moreover, TRIF also activates MyD88-independent NF-*κ*B activation by recruiting the adapter receptor-interacting protein 1 (RIP1).^58^ Furthermore, RIP1 can bind to TRIF, which causes both Fas-associated death domain-dependent apoptosis and NF-*κ*B activation.^14^ Thus, TRIF activates IRFs by interacting with TBK1 and IRAK1 and activates NF-*κ*B by interacting with RIP1 (Figures 2a and b).

## Involvement of TLR4 in Myocardial Inflammation

### Myocarditis

Current evidence has demonstrated that the host immune response and inflammation mainly mediate myocarditis, where TLR4 participates in the induction of pro-inflammatory and antiviral cytokines.

#### Viral myocarditis (VMC)

Viral infection is thought to be the most common cause of myocarditis, a leading pathogen of which can be Coxsackievirus B3 (CVB3). There is compelling evidence for the contribution of the inhibition of TLR4 system to ameliorating myocardial inflammation in VMC. In a mouse model of CVB3 infection, Fairweather *et al.* reported that TLR4-deficient mice are more resistant to CVB3 infection with decreased inflammatory responses, viral replication, and myocarditis compared with wild-type mice.^18^ CVB3 infection increases the cardiac levels of IL-1*β* and IL-18 in wild type but not TLR4-deficient mice. In contrast, Riad *et al.* reported that TRIF-deficient mice display a TLR4-dependent suppression of antiviral cytokine IFN-*β*, which can lead to the induction of severe heart failure and 100% mortality.^59^ Notably, MyD88 knockout leads to reduced production of pro-inflammatory cytokines, such as IL-1*β* and IL-18, but increased expression of IRF3 and IFN-*β*, which shows a dramatically higher survival rate than wild-type mice.^60^ Thus, TLR4 can not only augment the severity of VMC through the MyD88-dependent pathway but can also limit the severity of VMC through the MyD88-independent pathway. However, the MyD88-dependent pathway has a more leading role than the MyD88-independent pathway in most cases of VMC.

#### Autoimmune myocarditis (AM)

AM is a T-helper 17-mediated autoimmune cardiac disease characterized by inflammatory infiltration that mostly involves lymphocytes and macrophages.^61^ It remains poorly understood what effect the effector T cells and inflammatory macrophages may exert in the course of AM. In particular, the role of TLR4 in AM has been controversially discussed.

Reportedly, TLR4 induces the synthesis of pro-inflammatory cytokines in DCs, such as IL-6, and leads to Th-17 differentiation and exacerbates the myocardial inflammation.^62^ In addition, Jenke and colleagues revealed that adiponectin administration attenuates inflammatory activation and interaction of cardiac and immune cells by diminishing TLR4 signaling pathways, thereby exerting a cardioprotective effect in AM.^63^ However, there is currently no hard evidence that TLR4 inhibition may result in beneficial effects on clinically meaningful end points. Interestingly, Bachmaier *et al.* studied a mouse experimental AM model and reported that the administration of the TLR4 agonist LPS significantly decreases the severity and prevalence of AM and reduces the number of autoantigen reactive effector T cells.^61^ This suggests that the innate immunity and inflammatory responses might also confer beneficial effects by activating cardioprotective signaling pathways via TLR4 signaling.

Therefore, myocardial inflammation is a generic process. It is not always clear whether inflammation is beneficial or detrimental since there is a broad spectrum of inflammatory reactions. From an evolutionary perspective, it is likely that the fundamental role of TLR4 is to protect the heart against DAMPs, but on the other hand, TLR4 is also associated with deleterious inflammatory effects that exacerbate heart damage.

### MI

Although effective treatment of MI has greatly improved cardiac function and survival, MI is still a major cause of morbidity and mortality around the world. Necrotic cardiac myocytes due to MI release a wide range of endogenous DAMPs, associated with significant TLR4 induction.^64^ Activation of TLR4 causes increased expression of pro-inflammatory cytokines, which can lead to inflammatory responses and additional damage to the already injured myocardium. Notably, the TLR4 signaling pathways correlate with infarct severity but not with the extent of inflammation.^65^

TLR4 and downstream gene expression profiles are upregulated in both infarcted and remote myocardium following MI.^4^ Several studies have shown that the inhibition of TLR4 signaling pathways is beneficial for alleviating inflammatory responses and additional damage to the already injured myocardium. For instance, the injection of lentivirus short hairpin RNA against TLR4 into the infarcted heart can significantly decrease infarct size and improve cardiac function *in vivo*.^4^ Besides, the TLR4 inhibitor metformin leads to minor expression of mediators implicated in MI and damage, including TNF-*α*, IL-6, and IL-10.^19^

Several studies explored the multiple DAMPs released from necrotic cardiac myocytes that activate TLR4 following MI. S100 calcium-binding protein A1 (S100A1) has been reported to be a TLR4 agonist and also an endogenous alarmin. Rohde *et al.* reported that S100A1 is induced in mice with MI.^66^ Intracardial injection of S100A1 enlarges infarct size and worsens left ventricular functional performance post-MI.^66^ S100A8/A9, also an endogenous ligand of TLR4, is released following MI and amplifies the inflammatory responses.^67^ In addition, HMGB1, galectin-3, S100*β*, and IL-1*α* released by necrotic cardiac myocytes can induce myocardial inflammation through TLR4 signaling pathways.^68^ Thus, DAMPs and TLR4 might be useful biomarkers and therapeutic targets in myocardial inflammation.

Although TLR4 signaling pathways classically induce NF-*κ*B activation and further lead to the expression of pro-inflammatory genes, a recent study showed that patients with ST-segment elevation MI have increased the expression of a series of genes that implicate NF-*κ*B activity, including hypoxia-inducible factor-1*α* (HIF-1*α*), NF-*κ*BI*α*, IL-18R1/2, matrix metalloproteinase 9, and IL-8, but reduced the expression of TLR4-induced genes, such as TNF-*α*.^69^ Hence, besides the two classical TLR4 signaling pathways, there might be a novel HIF-1-dependent non-canonical TLR4 signaling pathway, the precise mechanisms of which have not been determined. Further studies are still required to devise methods to protect the myocardium from additional damage and to contribute to the treatment of MI.

### Myocardial I/R injury

Recent studies using mouse myocardial I/R injury models have revealed that TLR4 and NF-*κ*B expression levels are significantly increased in both the ischemic zone and the potential danger region and that cardiac myocyte apoptosis is induced during the early period following I/R injury.^70, 71^ Interestingly, during the late period, TLR4 and pro-inflammatory cytokine levels rise significantly again, which remodels the ventricular myocardium and affects the structure and function of the injured myocardium.^72^

Numerous studies have demonstrated that inhibiting TLR4 signaling pathways can attenuate inflammatory responses and cardiac myocyte apoptosis following myocardial I/R injury. Wang and colleagues reported that pterostilbene decreases TNF-*α* production via suppressing TLR4-NF-κB signaling pathways, which effectively inhibits the infiltration of neutrophils and subsequently attenuates inflammatory responses and cell apoptosis following myocardial I/R injury.^73^ Furthermore, cytokines that can be increased by TLR4 and its related pathways, such as TNF-*α* and IL-6, regulate the apoptosis of cardiac myocytes.^74^

Promising findings reveal that some ligands of TLR4 or its downstream genes could be used as pre-conditioning inducers, which would enhance resistance to severe myocardial I/R injury. The mice pretreated with intravenous injection of eritoran, a TLR4 antagonist, for 10 min before myocardial I/R show significantly reduced infarction.^75^ Similarly, pretreatment with follistatin, a binding protein downstream of TLR4, could significantly reduce apoptosis and infarcts of epithelial cells, endothelial cells, and cardiac myocytes due to myocardial I/R injury via inhibition of the MyD88-dependent pathway.^76^ These data suggest that TLR4 and its downstream genes may be potential therapeutic targets and that pre-conditioning with these molecules may decrease the morbidity and mortality associated with MI.

Interestingly, the levels of activated Akt are significantly increased in the myocardia of TLR4-deficient mice compared with wild-type mice after myocardial I/R injury,^77, 78^ suggesting that the PI3K/Akt signaling pathway may act as a compensatory regulator in myocardial inflammation. Hua and colleagues reported that administration of PI3K inhibitors completely abrogates cardioprotection in TLR4-deficient mice after I/R injury.^78^ In addition, Li and colleagues reported that glucan phosphate, a ligand of TLR4, may shift TLR4 signaling pathways from a predominant NF-*κ*B pathway to the PI3K/Akt signaling pathway, which serves a protective role in myocardial I/R injury.^79^ The related mechanisms may involve TLR4 stimulation that leads to tyrosine phosphorylation of the TIR domain. The TIR domain subsequently disassociates from MyD88, binds the p85 regulatory subunit of PI3K, and phosphorylates Akt. Afterwards, activated Akt promotes survival and inhibits the apoptosis of cardiac myocytes. These data strongly indicate that there is reciprocal regulation between the NF-*κ*B and PI3K/Akt signaling pathways during myocardial I/R injury.

## The Clinical Prospective of TLR4 Inhibition

To enable the potential of TLR4 signaling pathways as therapeutic targets to be realized, regulatory and scientific issues concerning both basic and clinical development require diligent consideration. Currently, the unresolved key question is whether it is possible to attenuate deleterious myocardial inflammation and preserve the advantages of innate immunity at the same time during the regulation of TLR4 signaling. Fortunately, although sustaining TLR4 activation may have dangerous effects on innate immunity and lead to deleterious myocardial inflammation, there still exist several approaches to the negative regulation of TLR4 to counteract those effects. For instance, exploring the clinical role of immune suppression might be contributive to attenuating TLR4-mediated myocardial inflammation. Moreover, further studies should also facilitate the development of new therapies directed at the innate immunity components in myocardial inflammation. The potential of these approaches is illustrated by the development and application of a series of antagonists and inhibitors of TLR4, some of which have already been researched in the hearts of animals. Representative clinical trials targeting TLR4 and related pathways in myocardial inflammation have been fully summarized in the table attached to this review (Table 2).

One of the TLR4 antagonists, eritoran, is reported to be effective in an *in vivo* murine model of transverse aortic constriction-induced cardiac hypertrophy, as shown by decreased production of pro-inflammatory cytokines such as IL-1*β* and IL-6 and increased production of anti-inflammatory cytokines such as IL-10.^80^ In addition, radioprotective 105 (RP105), a TLR4 homolog that lacks the TIR domain and competitively inhibits TLR4 signaling, has shown promise in the treatment of cardiac dysfunction after MI. RP105 can hamper the role of TLR4 signaling pathways in post-infarction remodeling and confer protective effects on cardiac function after MI.^81^

However, the prevention or treatment of cardiac diseases with TLR4 inhibitors or antagonists to date has not been launched in human clinical trials. Still, the clinical applications of TLR4 inhibition have been attempted in other systems or organs. Kanzler *et al.* revealed that the most obvious role of a TLR4 antagonist is to inhibit the recognition of LPS by TLR4, the key triggering event in Gram-negative bacterial sepsis.^82^ Though the drug E5564 (eritoran) showed well toleration in phase II clinical trials of the cure for sepsis, it did not result in an ideal reduction of 28-day mortality in phase III clinical trial.^83, 84^ Multiple possible aspects might explain why eritoran administration failed to demonstrate a significant effect in phase III clinical trial. For instance, this study was designed to capture patients in the early stages of sepsis-induced organ dysfunction, but did not select patients with established severe sepsis as before. Besides, there are differential outcomes compared with the phase II trial in gram-positive bacterial sepsis, and future trials with eritoran or other MD2-TLR4 inhibitors should proceed with caution if patients with Gram-positive infections are included in the study population.

Furthermore, the TLR4-specific inhibitor TAK-242 is reported to promote hematoma absorption and significantly improve neurologic deficits following intracerebral hemorrhage in spite of the unknown target of the pathway.^85^ Another novel TLR4 antagonist Ibudilast can suppress the production of pro-inflammatory cytokines such as TNF and IL-6 and induce the production of anti-inflammatory cytokine IL-10 in central nervous system and is undergoing phase II trials of opioid dependence.^86^ NovImmune's NI-0101 is a humanized monoclonal antibody for the treatment of acute and chronic inflammation still in preclinical phase. It binds specifically and selectively to an epitope on human TLR4 and interferes with the dimerization required for intracellular signaling and induction of numerous pro-inflammatory pathways.^87^ Apart from these, a viral protein-derived peptide OPN-401 is able to inhibit TLR4 signaling but still in preclinical development.^88^

**Figure 3:**
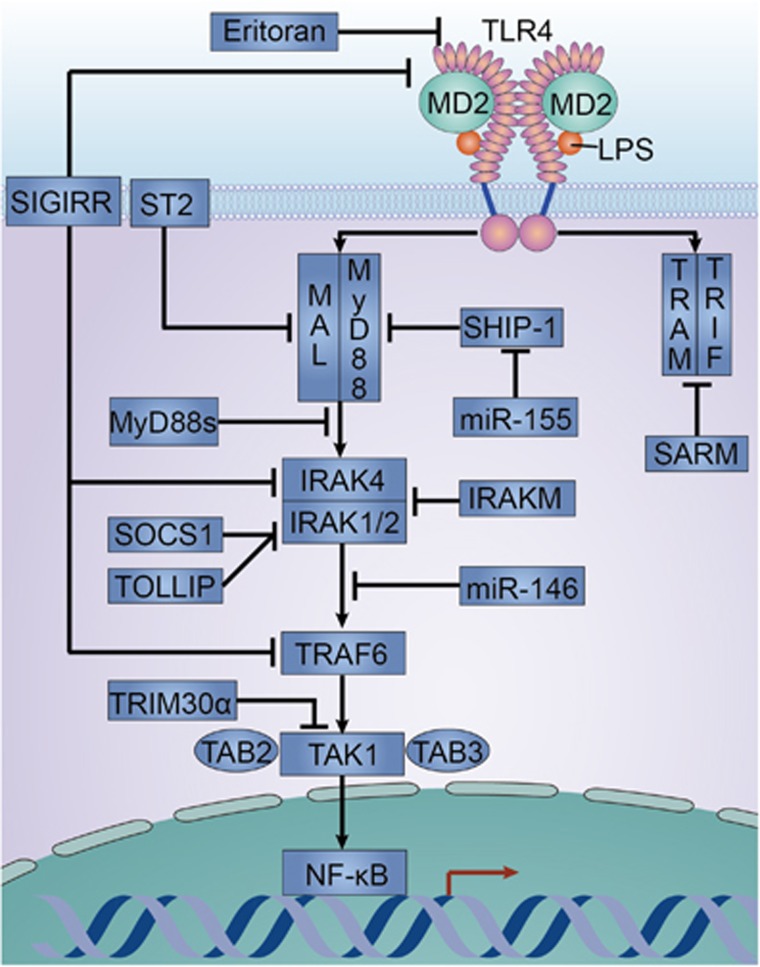
Negative regulation of TLR4 signaling pathways. The TLR4 antagonist eritoran inhibits TLR4 recognition of LPS. SHIP-1 inhibits the interaction between TLR4 and MyD88. ST2 sequesters MAL and MyD88. MyD88s can block the association between IRAK4 and MyD88. TOLLIP and SOCS1 can associate with IRAK1 and inhibit its activity. IRAKM inhibits the dissociation of IRAKs from MyD88 and prevents the formation of the IRAKs-TRAF6 complex. SIGIRR negatively regulates TLR4 signaling pathways by interacting transiently with TLR4, IRAK4, and TRAF6. TRIM30*α* destabilizes the TAK1 complex by promoting the degradation of TAB2 and TAB3. SARM specifically blocks the MyD88-independent signaling. miR-146 targets TRAF6 and IRAK1 and thus negatively regulates TLR4 signaling pathways. In addition, miR-155 can target SHIP-1 and thus suppress the negative regulation on TLR4 signaling pathways. Gene names: MAL, MyD88-adapter like; miR, microRNA; MyD88s, MyD88 short; SARM, Sterile *α*- and armadillo-motif-containing protein; SHIP-1, Src homology 2 domain-containing inositol 5-phosphatase 1; SOCS1, suppressor of cytokine signaling 1; ST2, suppression of tumorigenicity 2; TOLLIP, Toll-interacting protein

On the other hand, targeting the molecules of TLR4 downstream signaling pathways is also feasible. A variety of endogenous inhibitors that negatively regulate TLR4 downstream signaling pathways are reviewed (Figure 3).

## Conclusions

**Figure 4:**
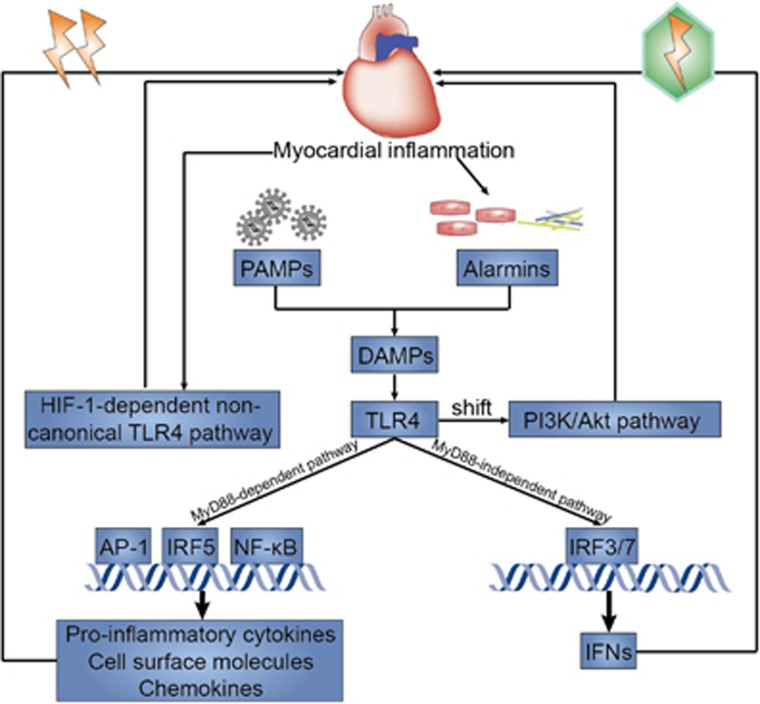
Graphical abstract. TLR4 recognizes and responds to DAMPs, including exogenous PAMPs and endogenous alarmins. The most common PAMPs in myocardial inflammation are thought to be virus, whereas alarmins released in response to myocardial inflammation can be necrotic cells and damaged matrix. TLR4 activates many transcription factors through the MyD88-dependent signaling pathway, subsequently inducing the production of pro-inflammatory cytokines, cell surface molecules, and chemokines and eventually exacerbating myocardial inflammation. TLR4 can also activate IRFs through the MyD88-independent signaling pathway, which further induce IFN production and ultimately exert an antiviral effect on myocardial inflammation. Moreover, a shift from predominant TLR4-NF-*κ*B signaling pathway to the PI3K/Akt signaling pathway may serve a protective role in myocardial inflammation. Furthermore, there might be a novel HIF-1-dependent non-canonical TLR4 signaling pathway during myocardial inflammation. IRF, IFN-regulatory factors; PI3K, phosphatidylinositol 3-kinase

This review elaborates on the emerging roles of TLR4 in myocardial inflammation, including myocarditis, MI, myocardial I/R injury, and heart failure (Figure 4). In summary, increasing lines of evidence suggest that TLR4 initiates the expression of a number of pro-inflammatory genes, cell surface molecules, and chemokines through the MyD88-dependent pathway, which exacerbates the damage to myocardium. Inhibiting TLR4 signaling pathways appears to be a promising method for alleviating myocardial inflammation. Moreover, pretreatment that targets TLR4 signaling pathways might effectively protect the myocardium from damage.

However, direct inhibition of TLR4 may not be a promising therapeutic avenue to improve the prognosis of myocardial inflammation to some extent, since TLR4 inhibition may lead to a functional loss of the innate immune mechanism. TLR4 can also play a beneficial role in myocardial inflammation, especially in myocarditis. TLR4 activates the MyD88-independent pathway and further promotes IFN-*β* expression, exerting an antiviral effect on myocardial inflammation. Thus, although excessive pro-inflammatory cytokine production exerts detrimental effects in myocardial inflammation, some cytokines also drive the host defense and tissue repair process within the heart.

Notably, some other signaling pathways can crosstalk with the TLR4 signaling pathways and exert regulatory effects on myocardial inflammation. The PI3K/Akt signaling pathway may serve as an endogenous negative feedback regulator of TLR4 signaling in myocardial inflammation and contribute to less maladaptive remodeling in TLR4-deficient mice. A shift from predominant TLR4-NF-*κ*B signaling pathway to the PI3K/Akt signaling pathway may serve a protective role in myocardial inflammation. Apart from the classical TLR4 and related signaling pathways, there might be a novel HIF-1-dependent non-canonical TLR4 signaling pathway during myocardial inflammation, as evidenced by an increase in the expression of a series of genes that implicate NF-*κ*B activity, such as HIF-1*α*, and a decrease in the expression of TLR4-induced genes, such as TNF-*α*.

Despite substantial progress, there remain many unresolved questions as to the complete transition towards clinical application, which are critical for our comprehensive understanding of the roles of TLR4 signaling pathways. For example, what is the exact role of TLR4 in the pathogenesis of cardiac diseases, pro-inflammatory or anti-inflammatory? Detrimental or protective? How would immune suppression influence TLR4 system? What are the specific TLR4 inhibitors peculiar to myocardial inflammation? How could the innate immunity components be clinically targeted? These questions will have to be settled before TLR4 can be considered a valuable therapeutic target for myocardial inflammation.

## Figures and Tables

**Table 1:** Exogenous and endogenous ligands and localization of TLRs

**TLRs**	**Exogenous ligands**	**Endogenous ligands**	**Localization**	**Signaling**
TLR1	Triacyl-lipopeptide		Cell surface	MAL-MyD88-NF-*κ*B/AP-1/IRF5 pathway
TLR2	Lipoproteins/lipopeptides, peptidoglycan, LTA, lipoarabinomannan, glycosylphosphatidylinositol anchors, zymosan, glycolipids, and porins	HSP 60, HSP 70, Gp96, and saturated fatty acids	Cell surface	MAL-MyD88-NF-*κ*B/AP-1/IRF5 pathway; MAL-MyD88-NF-*κ*B/AP-1 pathway
TLR3	dsRNA	mRNA	Intracellular compartments	PI3K/TRIF-IRF3 pathway; TRAM-TRIF-NF-*κ*B pathway; PI3K/TRIF-RIP1-NF-*κ*B pathway
TLR4	LPS, RSV protein F, MMTV envelope protein, VSV glycoprotein G, mannan, glucuronoxylomannan, glycosylinositolphospholipids, and paclitaxel	Biglycan, CD 138, crystallin A chain, *β*-defensin 2, fibrinogen, fibronectin, heparan sulfate, HMGB1, HSP22–60–70–72, hyaluronan, monosodium urate crystals, oxPAPC, resistin, surfactant protein A, tenascin-C, *β*-amyloid, gp 96, OxLDL, AGE-LDL, angiotensin II, calprotectin, ceramide, mmLDL, MRP-8/14, PAUF, serum amyloid A, and SFA	Cell surface	MAL-MyD88-NF-*κ*B/AP-1 pathway; TRAM-TRIF-NF-*κ*B/IRF3/IRF7 pathway
TLR5	Flagellin		Cell surface	MyD88- NF-*κ*B/IRF5 pathway
TLR6	Phenol-soluble modulin, diacyl, lipopeptides, LTA, zymosan, oxLDL, amyloid-*β* fibrils		Cell surface	Mal-MyD88-NF-*κ*B/AP-1 pathway
TLR7	ssRNA, imidazoquinoline, loxoribine, bropirimine, resiquimod, and imiquimod		Intracellular compartments	MyD88 and endosomal acidification (maturation)-IRF7 pathway; MyD88- NF-*κ*B pathway
TLR8	ssRNA, resiquimod		Intracellular compartments	MyD88 and endosomal acidification (maturation)-IRF7 pathway; MyD88- NF-*κ*B pathway
TLR9	Unmethylated CpG DNA, haemozoin	Chromatin-IgG complexes	Intracellular compartments	
TLR10		Unknown		
TLR11	Profilin, uropathogenic *E. coli*		Cell surface	

**Table 2:** Representative clinical trials that targeting TLR4 and related pathways in myocardial inflammation

**Diseases**	**Treatment**	**Effects**
AM	APN	Inhibition of TLR4-MyD88-NF-*κ*B-TNF-*α*/IL-6 and MyD88-independent apoptosis pathways^63^
AM	LPS	Induction of TLR4 and production of the anti-inflammatory chemokine CXCL1/KC^61^
MI	Metformin	Suppression of TLR4-MyD88-NF-*κ*B-TNF-*α*/IL-6 pathway^19^
Myocardial I/R injury	Pterostilbene	Suppression of TLR4-NF-∂B-TNF-*α* pathway, NO production, and attenuation of inflammatory responses and cell apoptosis^73^
Myocardial I/R injury	AsIV	Downregulation of TLR4-NF-*κ*B-TNF-*α*/IL-1*β* pathway and inhibition of cell apoptosis^70^
Myocardial I/R injury	Eritoran	Pretreatment with eritoran suppresses TLR4-MyD88-TRAF6-TAK1-JNK and TLR4-MyD88-TRAF6-TAK1-IKKs-NF-∂B pathways and further decreases expression of TNF-*α*, IL-1*β*, and IL-6^75^
Myocardial I/R injury	Follistatin	Pretreatment with follistatin inhibits the TLR4-MyD88-dependent pathway and reduces apoptosis and infarcts of cardiac myocytes^76^
Myocardial I/R injury	GP	A shift from a predominant TLR4-NF-*κ*B pathway to the PI3K/Akt pathway; cardioprotective effects^79^

Abbreviations: AM, autoimmune myocarditis; APN, adiponectin; AsIV, astragaloside IV; GP, glucan phosphate; IL, interleukin; IKK, inhibitory *κ*B kinase; I/R, ischemia/reperfusion; JNK, c-Jun N-terminal kinase; LPS, lipopolysaccharide; MI, myocardial infarction; MyD88, myeloid differentiation factor 88; NF-*κ*B, nuclear factor-*κ*B; NO, nitric oxide; PI3K, phosphatidylinositol 3-kinase; TLR4, Toll-like receptor; TNF-*α*, tumor necrosis factor-*α*; TRAF6, tumor necrosis factor receptor-associated factor 6; TAK1, transforming growth factor-*β*-activated kinase 1

## Abbreviations

AP-1: activator protein-1
AM: autoimmune myocarditis
CVB3: Coxsackievirus B3
DAMP: damage-associated molecular pattern
HIF-1: hypoxia-inducible factor-1
HMGB1: high mobility group box 1
IFN: interferon
I*κ*B: inhibitory *κ*B
I/R: ischemia/reperfusion
IRAK: interleukin-1 receptor-associated kinase
IRF: interferon-regulatory factor
LBP: lipopolysaccharide binding protein
LPS: lipopolysaccharide
LRR: leucine-rich repeat
MAPK: mitogen-activated protein kinase
MD2: myeloid differentiation factor 2
MI: Myocardial infarction
mRNA: messenger RNA
MyD88: myeloid differentiation factor 88
NF-*κ*B: nuclear factor-*κ*B
PAMP: pathogen-associated molecular pattern
PRRs: pattern recognition receptors
RIP1: receptor-interacting protein 1
RP105: radioprotective 105
SNP: single nucleotide polymorphisms
SR: scavenger receptor
TAB: transforming growth factor-*β*-activated kinase 1-binding protein
TBK1: TRAF family member-associated NF-*κ*B activator-binding kinase 1
TIR: Toll/interleukin-1 receptor
TLR: Toll-like receptor
TNF: tumor necrosis factor
TRAF: tumor necrosis factor receptor-associated factor
TRAM: TRIF-related adapter molecule
UBC13: ubiquitin-conjugating enzyme 13
UEV1A: ubiquitin-conjugating enzyme variant 1A
VMC: viral myocarditis
